# Case Report: From disordered eating to an eating disorder—a case study of an orienteering athlete with anorexia nervosa and the shortcomings of the multidisciplinary approach

**DOI:** 10.3389/fpsyg.2025.1537844

**Published:** 2025-03-14

**Authors:** Réka Erika Kovács, Szilvia Boros

**Affiliations:** ^1^Doctoral School of Education, Faculty of Pedagogy and Psychology, Eötvös Loránd University, Budapest, Hungary; ^2^National Institute for Sports Medicine, Budapest, Hungary; ^3^Department of Health and Nursing Sciences, Faculty of Health and Sport Sciences Széchenyi István University, Győr, Hungary

**Keywords:** disordered eating, eating disorder, athlete, anorexia nervosa, multidisciplinary approach

## Abstract

This case study explores the transition from disordered eating (DE) to an eating disorder (ED) in a 23-year-old female orienteer. Despite her talent as an athlete, her eating habits and training practices led to significant health concerns. After following an ovo-lacto vegetarian diet for 3 years, she exhibited symptoms of DE, including low energy intake (1,200 kcal/day), low body weight (50.1 kg, BMI: 16.9), and amenorrhea. Her condition deteriorated over 2 years, resulting in a diagnosis of anorexia nervosa (AN) by February 2023. During the treatment process, the athlete utilized a multidisciplinary approach that included dietitians, psychologists, and physicians. Despite achieving some initial progress, including a slight increase in body weight and the return of menstruation in July 2022, her health declined after psychological consultations were halted, leading to a further decrease in body fat and persistent low serum iron levels. This case highlights the importance of continuous monitoring, timely intervention, and a coordinated multidisciplinary team in addressing DE and ED in athletes. It also highlights the significance of effective communication among healthcare professionals and the need for comprehensive treatment strategies that include psychological, nutritional, and medical support. This study highlights the significance of early detection, suitable intervention, and the prevention of long-term health complications, such as decreased bone density and cardiovascular issues, in athletes with DE and ED.

## Introduction

Adequate nutrient intake is essential for achieving optimal athletic performance (Holtzman and Ackerman, 2021; Jeukendrup, 2017). Individualized nutrition strategies, developed by dietitians and sports nutritionists, play a crucial role in health promotion, body composition optimization, performance enhancement, and postinjury recovery (Chatterton and Petrie, 2013; Martinsen and Sundgot-Borgen, 2013; Sundgot-Borgen and Torstveit, 2004). However, many athletes depend on unqualified sources—such as coaches, parents, or teammates—for nutrition advice. This can lead to unsupervised practices that increase the risk of disordered eating (DE) and eating disorders (ED) (Mancine et al., 2020).

The prevalence of DE and ED among athletes is rising and is notably higher compared to the general population (Ghazzawi et al., 2024; Martinsen and Sundgot-Borgen, 2013; Sundgot-Borgen and Torstveit, 2004). While it was once believed that only female atheletes suffer from DE and ED, it is now recognized that male counterparts can also be affected (Chatterton and Petrie, 2013). However, female athletes still exhibit a higher prevalence of these conditions (Hazzard et al., 2020; Cabre et al., 2022). Every sport discipline is affected, but those involving esthetics (dance, gymnastics, rhythmic gymnastics, etc.), endurance (running, canoeing, diving, etc.), and weight-dependent sports (wrestling, judo, taekwondo, karate, etc.) are particularly at risk (Mancine et al., 2020; Sundgot-Borgen and Garthe, 2011). Additionally, team sports have been recognized as including a group of vulnerable athletes (Kampouri et al., 2019; Gouttebarge et al., 2018).

Among athletes, DE includes all pathological behaviors aimed at periodically reducing body weight, such as meal restriction, the use of weight-loss pills, binge eating, and purging, or enhancing sports performance (Wells et al., 2020). Clinical EDs are characterized by more severe and frequent behaviors, such as fasting, binge eating, and purging several times a week. Unlike DE, which involves a less pervasive preoccupation with food, ED significantly disrupts an individual’s daily life (Wells et al., 2020).

DE and ED are multifactorial conditions that exist on a spectrum, as described by the Australian Institute of Sport (AIS) and the National Eating Disorders Collaboration (NEDC) in 2020 (Wells et al., 2020). Optimal nutrition serves as the foundation, supplying the energy necessary for both health maintenance and athletic performance. When this balance is disrupted, symptoms of DE may appear. Without intervention, DE can progress to ED, a clinical condition with serious health implications (Wells et al., 2020).

Both DE and ED can be managed using a multidisciplinary approach, involving physicians, psychiatrists, and dietitians in the medical team (Cena et al., 2022; Temme and Hoch, 2013; Joy et al., 2003). Physicians assess overall health, prescribe medication when necessary, and suggest mental health and nutritional interventions (Mairs and Nichols, 2016; Woodruff et al., 2020; Cena et al., 2022). Dietitians examine eating behaviors, monitor physical symptoms, and develop personalized nutritional plans, while psychologists and psychiatrists provide psychotherapy, track mental health progress, and prescribe medication as needed. The multidisciplinary team may also involve physiotherapists, coaches, family members, teammates, or others essential to the athlete’s recovery (Joy et al., 2016). Despite this structured approach, recovery outcomes remain challenging, with only 40–50% of patients achieving full recovery (Joy et al., 2003).

There are several internationally accepted manuals for diagnosing EDs, such as the *Diagnostic and Statistical Manual of Mental Disorders* (DSM) (American Psychiatric Association, 2013), the *International Statistical Classification of Diseases and Related Health Problems* (ICD) (World Health Organization, 2019), the *Chinese Classification of Mental Disorders* (CCDM) (Chen, 2020), *Psychodynamic Diagnostic Manual* (PDM) (Lingiardi and McWilliams, 2015). Currently, *the Diagnostic and Statistical Manual of Mental Disorders*, Fifth Edition, Text Revision (DSM-V-TR) with text revisions is the most comprehensive clinical resource available including updated diagnostic criteria and the International Classification of Diseases, Tenth Revision, Clinical Modification (ICD-10-CM) codes (American Psychiatric Association, 2024).

Insufficient nutrient intake can negatively affect various areas. Among athletes, prolonged low energy availability (LEA), lasting for weeks or months, can lead to a decrease in sports performance as well as health deterioration, as defined by the term Relative Energy Deficiency in Sport (REDs) by the International Olympic Committee’s Medical Commission (Mountjoy et al., 2023). This syndrome affects multiple organ systems, including the endocrine system, cardiovascular system, immune system, and gastrointestinal system. It also impacts growth, bone health, and mental state (Cabre et al., 2022; Logue et al., 2020). Furthermore, the increased risk of injury contributes to the number of missed training hours, subsequently resulting in a decline in sports performance (Henninger et al., 2024; Joubert et al., 2020; Rauh et al., 2010).

Petrie and Greenleaf (2007) summarized a theoretical model highlighting several factors that may influence athletes’ disordered eating behaviors. This model suggests that social and sport-related pressures can lead to body dissatisfaction, which may result in restrictive eating, followed by binge eating and the onset of other disordered eating behaviors. Different moderators play a role at each stage, with body dissatisfaction influenced by weight and body shape, while restrictive eating is shaped by perfectionism, body perception, neuroticism, and self-esteem. Family and peer behavior models also play a role in the occurrence of the problem (Petrie and Greenleaf, 2007).

Although longitudinal studies on the connection between DE and ED are limited, research indicates that DE rarely resolves without appropriate intervention (Mountjoy et al., 2014; Gouttebarge and Kerkhoffs, 2017). Both DE and ED have negative effects on health in various ways, including adverse changes in body composition (reduced body weight and fat ratio) and abnormal blood test results (serum iron, ferritin, fasting blood sugar, and hormonal imbalances). These complications can result in long-term health issues, such as amenorrhea and iron deficiency, which hinder performance (Attwell et al., 2022; Hulmi et al., 2017; Banfi et al., 2012; Gordon, 2010). The diagnosis of EDs is often delayed because symptoms can remain hidden for extended periods (Tan et al., 2016).

All of these conditions underline the importance of early recognition and prevention, as well as the need for continuous joint action of the multidisciplinary team. In our study, there was a lack of proper cooperation among the members of the medical staff which led to serious health deterioration.

### Patient information

We present the 2-year case study of a 23-year-old female orienteer who has been involved in the sport since the age of 13 and has been following an ovo-lacto vegetarian diet for 3 years. She is a talented athlete; in the year prior to the intervention, she competed in 45 events and won medals in 20 of them. We aim to illustrate how her symptoms transitioned from DE to ED. This case study was approved by the Human Ethics Committee of ELTE Eötvös Loránd University, Faculty of Pedagogy and Psychology (license number: 2023/104). The athlete gave written permission to publish the results. Detailed first-year information is available from a previous study (Kovács and Boros, 2024). We sought special permission to disclose the data in the medical documentation from the institution where the treatments were conducted.

The starting point was in February 2022, when she visited the ambulatory dietetic care. She sought support from her coach and was also motivated by her own determination. Her current goal was to focus on achieving her best performance; however, her coach was rather worried about her health. According to her food diary, she consumed an average of 1,200 kcal/day (25.9 kcal/fat-free kgs) and trained 6 days per week. She was 1.73 meters tall, weighed 50.1 kg, had a BMI of 16.9 kg/m^2^, and a body fat percentage (PBF) of 7.5%. She had not had her menstrual period and had not undergone a gynecological examination since 2020. The laboratory results confirmed low levels of serum iron. Before she became vegetarian, she weighed 57–58 kgs, and her periods were regular.

### Therapeutic intervention and clinical findings

Following the nutritional assessment, she underwent consultations with an internist, psychologist, and psychiatrist, after which a common therapeutic assessment commenced in May 2022. According to the DSM-V, she did not meet the criteria of an ED, but she exhibited several DE symptoms. She counted calories but was not afraid of gaining weight; rather, she worried about not consuming a sufficient amount of energy. Moreover, she declined restaurant invitations because she believed that there would not be suitable dishes for vegetarians. Based on the medical team’s decision, it was not necessary to revoke her competition license or reduce her training volume. Psychiatric medicine prescription and hospitalization were also deemed unnecessary. She started working with a sports psychologist, who also worked with the athlete’s teammates. To help regulate her serum iron levels, Krauterblut syrup was recommended by the nutritionist (OGYI-NYTSZ-337/92).

Throughout the treatment process, monthly dietary assessments, biweekly psychological evaluations, and semiannual internal medicine examinations, including blood test analysis, were conducted. During the dietary consultations, body composition measurements [performed with an (InBody Co., Ltd., Seoul, Korea) device; Brewer et al., 2021] and food diary analysis (with Nutricomp DietCAD software) were conducted. The athlete did not want to follow a structured diet prepared by the specialist, but she agreed to discuss and review her meals during each meeting. The therapeutic goals were defined as follows: a minimum BMI of 18.5 kg/m^2^ (~ 55 kg), a minimum of 12% body fat, and the maintenance of regular menstrual cycles. The medical team never convened a joint meeting to discuss the athlete’s condition and further treatment recommendations, so the practitioners were informed by the athlete about the progress she was making in each of the respective fields. The internist and the dietitian were at the same institute, so they accessed each other’s documentation regarding the athlete; however, there was no information about the psychological consultations.

**Figure 1:**
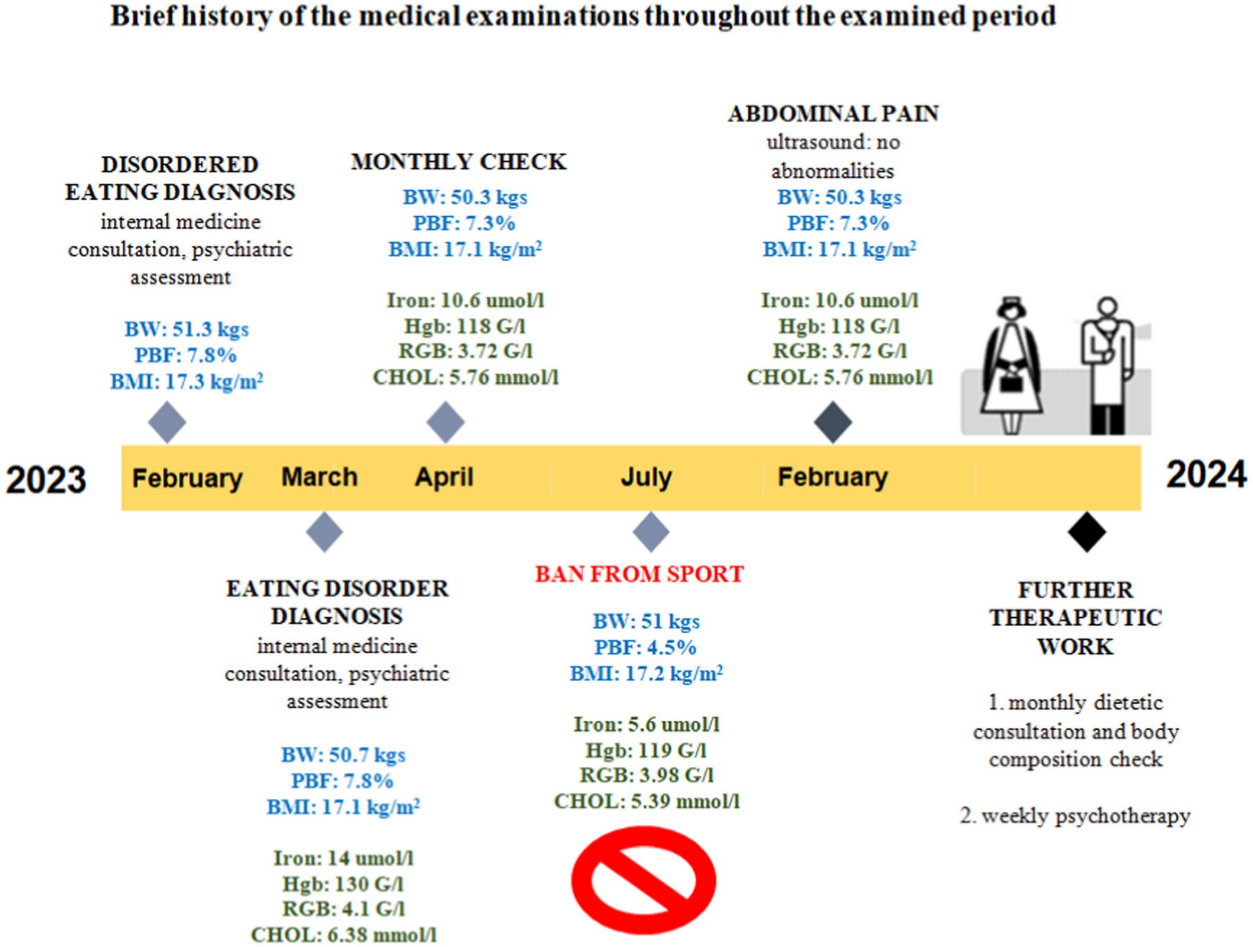
Brief history of the medical exaninations provided througout the examined period. Hgb, hemoglobin; RBC, red blood cell; CHOL, cholesterol; BW, body weight; PBF, percent body fat; BMI, body mass index; GOT, glutamate-oxalacetate-transaminase; GPT, glutamate-piruvate-transaminase; Gamma GT, gamma-glutamil-transferase; LDH, lactate-dehydrogenase.

In her first year, she achieved initial success. Her body weight slightly reached the therapeutic goal (54.8 kg), so her body fat percentage (11%), and in July 2022, she had her menstrual period. The body composition results started to deteriorate after the suspension of the psychological consultations (August 2022). By the end of the first year (February 2023), the orienteer was diagnosed with an ED [anorexia nervosa (AN)]. The events of the upcoming year are summarized in Figure 1. Psychological consultations were recommended to be restarted urgently, but this time weekly instead of biweekly. Concerning the competition license, she was still not banned from sport. The most important thing for the athlete was to be still able to compete, but she was not aware of her serious health condition. In April 2023, the athlete’s body weight dropped below 50 kgs (BMI 16.7 kg/m^2^). Her serum iron levels remained low, as did the red blood cell and hemoglobin concentrations. By July, the body fat percentage dropped to 4.5% when a cardiology examination was performed with no abnormalities. The medical team determined that AN clearly presented her medical history and current physical condition, which required the immediate revocation of her competition license. Hospitalization and psychiatric prescriptions were not recommended. At this point, the coach radically reduced the training volume rather than eliminating it entirely (as would have been necessary, but the athlete strongly opposed it). For the next 6 months, contact was made more difficult by an Erasmus scholarship program, which meant that the athlete was abroad. We overcame this by having her body composition checked at a foreign university, which was evaluated online by the dietitian. She also participated in individual online therapies with the sports psychologist. After returning home in February 2024, the athlete reported severe abdominal pain and spasms, especially after meals. An abdominal ultrasound was conducted, which revealed no abnormalities. However, the laboratory results indicated concerning findings for liver function (GOT-glutamate-oxalacetate-transaminase, GPT- glutamate-piruvate-transaminase, gamma GT- gamma-glutamil-transferase, LDH-lactate dehydrogenase), and no further investigation was conducted. It was recommended to start psychotherapy with a clinical psychiatrist specialized in eating disorders instead of a sports psychologist. From then on, therapy included weekly psychiatric consultations (now with the ED specialist), monthly dietary counseling, and quarterly internal medicine check-ups.

### Follow-up and outcomes

Since then, the athlete’s condition had improved. However, similar to the previous 2 years, there had been minor setbacks in body weight and body composition due to personal life problems. Her current weight was 54 kg, her body fat percentage was 10.8%, and her abdominal pain had disappeared. Although these values did not yet reach the target set at the beginning of the therapy, she had maintained them steadily in recent months. The frequency of psychiatric consultations had been reduced to once every 2 weeks. Psychiatric prescription was still not recommended.

In October 2024, after a joint decision by the medical team, the athlete was issued a 1-month competition license. Her training load had increased, but she had not yet returned to her preintervention level. The competition permit was renewed each month, with the consent of the dietitian and psychiatrist.

Regarding her eating habits, she continued to follow an ovo-lacto vegetarian diet. The blood test results indicated a slight increase in serum iron levels, hemoglobin, and red blood cells; however, these levels still remained low. Cholesterol and liver function markers had normalized. She has had her menstrual period once since then, marking the second occurrence since 2020. Consequently, the sports physician referred her for a dual-energy X-ray absorptiometry (DXA or DEXA) bone density test that revealed early-stage osteoporosis in the spine. Further endocrinological testing is ongoing.

## Discussion

### The relationship between low body weight, body fat percentage, and amenorrhea

The most serious issue in this case is the low body weight and body fat percentage, which was accompanied by amenorrhea. Low body weight, inadequate nutrition, and excessive physical activity can collectively disrupt the production of sex hormones (such as estrogen and luteinizing hormone), leading to menstrual irregularities or even the complete cessation of menstruation (Berz and McCambridge, 2016; Klein and Poth, 2013; Winston, 2012). This is particularly critical among female athletes, as amenorrhea not only threatens reproductive health but also raises risks, such as decreased bone density and stress fractures (Costa et al., 2022). Therefore, restoring energy balance and maintaining adequate body fat levels are essential (Melin et al., 2019; De Bruin, 2017; Mountjoy et al., 2014).

### The link between high cholesterol levels and anorexia nervosa

Throughout the observed period, the athlete’s cholesterol levels remained consistently elevated. Previous studies indicate that in cases of AN, endogenous cholesterol production increases, likely triggered by the body’s energy-deficient state (Hussain et al., 2019; Winston, 2012). This may arise from the liver’s compensatory mechanism trying to maintain the necessary lipid levels necessary for cellular function. This increase in cholesterol poses long-term cardiovascular risks, especially if other metabolic issues are present. A thorough review of the diet and addressing nutritional deficiencies are essential for managing the condition (Neglia, 2021; Melin et al., 2015).

### Suspected superior mesenteric artery syndrome

Although the abdominal ultrasound report was negative, the symptoms indicated the possibility of superior mesenteric artery syndrome (SMAS). This condition typically occurs in cases of significant weight loss, where the reduction of abdominal fat causes the artery to compress the duodenum, resulting in digestive issues and severe pain (Bloomberg et al., 2023; Singh and Contrucci, 2023). SMAS is often associated with EDs and can serve as a warning sign of AN (Recio-Barbero et al., 2019; Sato and Fukudo, 2015). Nevertheless, SMAS is a relatively rare condition; however, its incidence is higher in patients with EDs, especially AN, than in the general population (Farina et al., 2017; Oliva-Fonte et al., 2017).

### Challenges of vegetarianism for athletes

The athlete followed a vegetarian diet, which may be a challenge in restoring energy balance and adequate nutrient intake. However, numerous professional guidelines exist for the nutrition of vegetarian athletes, emphasizing that both health and performance enhancement can be achieved through proper planning (Craddock et al., 2016; Barr and Rideout, 2004). Nevertheless, these diets require careful attention, especially regarding adequate intake of essential proteins, iron, calcium, vitamin D, and vitamin B12 (Nebl et al., 2019; West et al., 2023). Collaborating with a sports dietitian is essential to ensure that the athlete’s diet meets the requirements for recovery and hormonal balance restoration (Kussman and Choo, 2024).

### Treatment recommendations and multidisciplinary approach

By the end of the first year, the athlete’s condition further deteriorated, necessitating the revocation of the competition license. This decision aligns with other case studies that indicate better long-term outcomes when competition is suspended, allowing for a focus on rehabilitation (Quatromoni, 2017).

A multidisciplinary approach is essential for effective treatment as recommended by the IOC (Mountjoy et al., 2023). Collaborating with a psychologist or psychiatrist who specializes in ED is crucial, as psychotherapy assists in uncovering and addressing the underlying psychological causes (Grilo, 2024; Eichstadt et al., 2020; Hay, 2020; Petisco-Rodríguez et al., 2020; Murphy et al., 2010). Furthermore, a controlled and gradually increased nutritional intake, along with a temporary halt to physical activity, is vital for the body’s recovery (Neglia, 2021).

This athlete’s situation shows that improvement is possible with appropriate treatment. The condition can be reversed when physical, nutritional, and psychological aspects are thoughtfully considered and addressed in an integrated approach.

### Limitations

The case study presents a detailed overview of an athlete’s journey. However, several limitations must be considered. This is a single case study, which means that the findings are specific to one individual. While it can provide valuable insights into the athlete’s experience, these results cannot necessarily be generalized to all athletes, particularly those with varying eating habits, training routines, or backgrounds. Although psychological treatments and interventions were part of the athlete’s care, we were unable to provide detailed information on the types of therapies used. Furthermore, an eating disorder specialist was involved only after a year and a half. The specific impact of these therapies on her recovery remains unclear. The athlete’s progress was monitored by various specialists (internist, dietitian, psychologist, and psychiatrist) who did not always collaborate directly. This lack of coordinated care may have influenced the outcomes and indicates that a more integrated, multidisciplinary approach might have been more beneficial.

## Conclusion

This case study highlights the need for developing effective preventive strategies to reverse the effects of disordered eating (DE) and to prevent the onset of eating disorders (EDs) in athletes. Early intervention is essential, and coaches play a vital role in identifying subtle shifts in an athlete’s behavior, eating habits, or physical performance. Their awareness can help prompt early medical evaluations, preventing further progression of DE. However, once DE symptoms are recognized, medical staff must make informed decisions about temporarily suspending training or competitions to prioritize the athlete’s physical and mental health.

Although a theoretical framework for multidisciplinary team care exists, practical challenges often hinder its implementation. Limited resources, time constraints, and challenges in coordinating professionals from diverse fields, each with unique priorities, can impede effective collaboration. This case study highlights the need for improvements in two key areas: early identification of at-risk athletes and better collaboration within multidisciplinary teams.

To address these challenges, developing more effective screening methods—such as regular check-ins or questionnaires for athletes and coaching staff— and emphasizing collaboration among medical team members—could provide early indicators of DE or ED.

## Data Availability

The original contributions presented in the study are included in the article/supplementary material, further inquiries can be directed to the corresponding author/s.

## References

[ref1] American Psychiatric Association (2013). Diagnostic and statistical manual of mental disorders: DSM-5. 5th Edn. Washington, DC: American Psychiatric Publishing.

[ref2] American Psychiatric Association (2024). Unrecognised Eating disorders in boys young men. Available at: https://www.apa.org/monitor/2024/10/eating-disorders-boys-men (Accessed February 28, 2025).

[ref3] Attwell C. Dugan C. McKay A. K. A. Nicholas J. Hopper L. Peeling P. (2022). Dietary Iron and the elite dancer. Nutrients 14:1936. doi: 10.3390/nu14091936 35565904 PMC9105128

[ref4] Banfi G. Colombini A. Lombardi G. Lubkowska A. (2012). “Metabolic markers in sports medicine” in Advances in clinical chemistry (Elsevier), 1–54. 10.1016/b978-0-12-394317-0.00015-7 22397027

[ref5] Barr S. I. Rideout C. A. (2004). Nutritional considerations for vegetarian athletes. Nutrition 20, 696–703. doi: 10.1016/j.nut.2004.04.015, PMID: 15212753

[ref6] Berz K. McCambridge T. (2016). Amenorrhea in the female athlete: what to do and when to worry. Pediatr. Ann. 45. doi: 10.3928/00904481-20160210-03, PMID: 27031318

[ref7] Bloomberg L. Hoscheit M. Hendler S. Abegunde A. T. (2023). Superior mesenteric artery syndrome in an adolescent female with anorexia nervosa. Clin. Med. Res. 21, 46–48. doi: 10.3121/cmr.2022.1768, PMID: 37130783 PMC10153676

[ref8] Brewer G. J. Blue M. N. M. Hirsch K. R. Saylor H. E. Gould L. M. Nelson A. G.. (2021). Validation of InBody 770 bioelectrical impedance analysis compared to a four-compartment model criterion in young adults. Clin. Physiol. Funct. 41, 317–325. doi: 10.1111/cpf.12700, PMID: 33752260

[ref9] Cabre H. Moore S. Smith-Ryan A. Hackney A. (2022). Relative energy deficiency in sport (RED-S): scientific, clinical, andpractical implications for the female athlete. Dtsch Z Sportmed 73, 225–234. doi: 10.5960/dzsm.2022.546, PMID: 36479178 PMC9724109

[ref9004] Cena H. Vandoni M. Magenes V. C. Di Napoli I. Marin L. Baldassarre P.. (2022). Benefits of exercise in multidisciplinary treatment of binge eating disorder in adolescents with obesity. IJERPH 19:8300. doi: 10.3390/ijerph19148300 35886152 PMC9315465

[ref10] Chatterton J. M. Petrie T. A. (2013). Prevalence of disordered eating and pathogenic weight control behaviors among male collegiate athletes. Eat. Disord. 21, 328–341. doi: 10.1080/10640266.2013.797822, PMID: 23767673

[ref11] Chen Y. F. (2020). Chinese classification of mental disorders (CCMD-3): towards integration in international classification. Psychopathology 35, 171–175. doi: 10.1159/000065140, PMID: 12145505

[ref12] Costa T. M. D. R. L. Borba V. Z. C. Correa R. G. P. Moreira C. A. (2022). Stress fractures. Arch Endocrinol Metabol 66, 765–773. doi: 10.20945/2359-3997000000562, PMID: 36382766 PMC10118812

[ref13] Craddock J. C. Probst Y. C. Peoples G. E. (2016). Vegetarian and omnivorous nutrition—comparing physical performance. Int. J. Sport Nutr. Exerc. Metab. 26, 212–220. doi: 10.1123/ijsnem.2015-0231, PMID: 26568522

[ref14] De Bruin A. P. (2017). Athletes with eating disorder symptomatology, a specific population with specific needs. Curr. Opin. Psychol. 16, 148–153. doi: 10.1016/j.copsyc.2017.05.009, PMID: 28813340

[ref15] Eichstadt M. Luzier J. Cho D. Weisenmuller C. (2020). Eating disorders in male athletes. Sports Health 12, 327–333. doi: 10.1177/1941738120928991, PMID: 32525767 PMC7787561

[ref16] Farina R. Foti P. V. Cocuzza G. Costanzo V. Costanzo G. Conti A.. (2017). Wilkie’s syndrome. J Ultrasound 20, 339–342. doi: 10.1007/s40477-017-0257-2, PMID: 29204239 PMC5698186

[ref17] Ghazzawi H. A. Nimer L. S. Haddad A. J.. (2024). A systematic review, meta-analysis, and meta-regression of the prevalence of self-reported disordered eating and associated factors among athletes worldwide. J. Eat. Disord. 12:24. doi: 10.1186/s40337-024-00982-5 38326925 PMC10851573

[ref18] Gordon C. M. (2010). Clinical practice. Functional hypothalamic amenorrhea. N. Engl. J. Med. 363, 365–371. doi: 10.1056/NEJMcp0912024, PMID: 20660404

[ref19] Grilo C. M. (2024). Treatment of eating disorders: current status, challenges, and future directions. Annu. Rev. Clin. Psychol. 20, 97–123. doi: 10.1146/annurev-clinpsy-080822-043256, PMID: 38211625

[ref9002] Gouttebarge V. Hopley P. Kerkhoffs G. Verhagen E. Viljoen W. Wylleman P.. (2018). A 12-month prospective cohort study of symptoms of common mental disorders among professional rugby players. EJSS, 18, 1004–1012. doi: 10.1080/17461391.2018.1466914 29698129

[ref9001] Gouttebarge V. Kerkhoffs G. M. M. J. (2017). A prospective cohort study on symptoms of common mental disorders among current and retired professional ice hockey players. PHSPDE 45, 252–258. doi: 10.1080/00913847.2017.1338497 28576114

[ref20] Hay P. (2020). Current approach to eating disorders: a clinical update. Intern. Med. J. 50, 24–29. doi: 10.1111/imj.14691, PMID: 31943622 PMC7003934

[ref9003] Hazzard V. M. Schaefer L. M. Mankowski A. Carson T. L. Lipson S. M. Fendrick C.. (2020). Development and validation of the eating disorders screen for athletes (EDSA): A brief screening tool for male and female athletes. Psychol Sport and Exer, 50:101745. doi: 10.1016/j.psychsport.2020.101745 PMC7392177 32733166

[ref21] Henninger K. Pritchett K. Brooke N. K. Dambacher L. (2024). Low energy availability, disordered eating, exercise dependence, and fueling strategies in trail runners. Int. J. Exerc. Sci. 16, 1471–1486. doi: 10.70252/FFDK5934 38288400 PMC10824294

[ref22] Holtzman B. Ackerman K. E. (2021). Recommendations and nutritional considerations for female athletes: health and performance. Sports Med. 51, 43–57. doi: 10.1007/s40279-021-01508-8, PMID: 34515972 PMC8566643

[ref23] Hulmi J. J. Isola V. Suonpää M. Järvinen N. J. Kokkonen M. Wennerström A.. (2017). The effects of intensive weight reduction on body composition and serum hormones in female fitness competitors. Front. Physiol. 7:689. doi: 10.3389/fphys.2016.00689, PMID: 28119632 PMC5222856

[ref24] Hussain A. A. Hübel C. Hindborg M. Lindkvist E. Kastrup A. M. Yilmaz Z.. (2019). Increased lipid and lipoprotein concentrations in anorexia nervosa: a systematic review and meta-analysis. Int. J. Eat. Disord. 52, 611–629. doi: 10.1002/eat.23051, PMID: 30920679 PMC6842568

[ref25] Jeukendrup A. E. (2017). Periodized nutrition for athletes. Sports Med. 47, 51–63. doi: 10.1007/s40279-017-0694-2, PMID: 28332115 PMC5371625

[ref26] Joubert L. M. Gonzalez G. B. Larson A. J. (2020). Prevalence of disordered eating among international sport Lead rock climbers. Front. Sports Act. Living 2:86. doi: 10.3389/fspor.2020.00086 33345077 PMC7739584

[ref9005] Joy E. Kussman A. Nattiv A. (2016). 2016 update on eating disorders in athletes: A comprehensive narrative review with a focus on clinical assessment and management. BJSM, 50, 154–162. doi: 10.1136/bjsports-2015-095735 26782763

[ref9014] Joy E. A. Wilson C. Varechok S. (2003). The multidisciplinary team approach to the outpatient treatment of disordered eating. Curr Sport Med Rep, 2, 331–336. doi: 10.1249/00149619-200312000-00009 14583163

[ref9006] Kampouri D. Kotopoulea-Nikolaidi M. Daskou S. Giannopoulou I. (2019). Prevalence of disordered eating in elite female athletes in team sports in Greece. EJSS, 19, 1267–1275. doi: 10.1080/17461391.2019.1587520 30880593

[ref27] Klein D. A. Poth M. A. (2013). Amenorrhea: an approach to diagnosis and management. Am. Fam. Phys. 87, 781–788. 23939500

[ref28] Kovács R. E. Boros S. (2024). Case study: an orienteer athlete with disordered eating. M Sporttud Szle 110, 34–37.

[ref9007] Kussman A. Choo H. J. (2024). Mental health and disordered eating in athletes. Clin Sports Med, 43, 71–91. doi: 10.1016/j.csm.2023.07.001 37949515

[ref29] Lingiardi V. McWilliams N. (2015). The psychodynamic diagnostic manual - 2nd edition (PDM-2). WPA 14, 237–239. doi: 10.1002/wps.20233, PMID: 26043343 PMC4471982

[ref30] Logue D. M. Madigan S. M. Melin A. Delahunt E. Heinen M. Donnell S.-J. M.. (2020). Low energy availability in athletes 2020: an updated narrative review of prevalence, risk, within-day energy balance, knowledge, and impact on sports performance. Nutrients 12:835. doi: 10.3390/nu12030835, PMID: 32245088 PMC7146210

[ref31] Mancine R. P. Gusfa D. W. Moshrefi A. Kennedy S. F. (2020). Prevalence of disordered eating in athletes categorized by emphasis on leanness and activity type – a systematic review. J. Eat. Disord. 8:47. doi: 10.1186/s40337-020-00323-2 33005418 PMC7523350

[ref9015] Mairs R. Nicholls D. (2016). Assessment and treatment of eating disorders in children and adolescents. Arch Dis Child, 101, 1168–1175. doi: 10.1136/archdischild-2015-309481 27381185

[ref32] Martinsen M. Sundgot-Borgen J. (2013). Higher prevalence of eating disorders among adolescent elite athletes than controls. Med. Sci. Sports Exerc. 45, 1188–1197. doi: 10.1249/MSS.0b013e318281a939, PMID: 23274604

[ref33] Melin A. K. Heikura I. A. Tenforde A. Mountjoy M. (2019). Energy availability in athletics: health, performance, and physique. Int. J. Sport Nutr. Exerc. Metab. 29, 152–164. doi: 10.1123/ijsnem.2018-0201, PMID: 30632422

[ref34] Melin A. Tornberg Å. B. Skouby S. Møller S. S. Sundgot-Borgen J. Faber J.. (2015). Energy availability and the female athlete triad in elite endurance athletes. Scand. Med. Sci. Sports 25, 610–622. doi: 10.1111/sms.12261, PMID: 24888644

[ref35] Mountjoy M. Ackerman K. E. Bailey D. M. Burke L. M. Constantini N. Hackney A. C.. (2023). 2023 International Olympic Committee's (IOC) consensus statement on relative energy deficiency in sport (REDs). Br. J. Sports Med. 57, 1073–1097. doi: 10.1136/bjsports-2023-106994, PMID: 37752011

[ref9008] Mountjoy M. Sundgot-Borgen J. Burke L. Carter S. Constantini N. Lebrun C.. (2014). The IOC consensus statement: beyond the female athlete triad—relative energy deficiency in sport (RED-S). BJSM, 48, 491–497. doi: 10.1136/bjsports-2014-093502 24620037

[ref36] Murphy R. Straebler S. Cooper Z. Fairburn C. G. (2010). Cognitive behavioral therapy for eating disorders. Psychiatr. Clin. N. Am. 33, 611–627. doi: 10.1016/j.psc.2010.04.004, PMID: 20599136 PMC2928448

[ref9009] Nebl J. Schuchardt J. P. Ströhle A. Wasserfurth P. Haufe S. Eigendorf J.. (2019). Micronutrient status of recreational runners with vegetarian or non-vegetarian dietary patterns. Nutrients, 11:1146. doi: 10.3390/nu11051146 31121930 PMC6566694

[ref37] Neglia A. (2021). Nutrition, eating disorders, and behavior in athletes. Psychiatr. Clin. North Am. 44, 431–441. doi: 10.1016/j.psc.2021.04.009, PMID: 34372999

[ref9010] Oliva-Fonte C. Fernández Rey C. Pereda Rodríguez J. González-Fernández A. M. (2017). Wilkie´s syndrome. Rev Espan Enferm Dis, 109, 62–63. 28100057

[ref38] Petisco-Rodríguez C. Sánchez-Sánchez L. C. Fernández-García R. Sánchez-Sánchez J. García-Montes J. M. (2020). Disordered eating attitudes, anxiety, self-esteem and perfectionism in young athletes and non-athletes. IJERPH 17:6754. doi: 10.3390/ijerph17186754, PMID: 32948005 PMC7559299

[ref39] Petrie T. A. Greenleaf C. A. (2007). Eating disorders in sport: from theory to research to intervention. In G. In Tenenbaum G. Eklund R. C. (Eds.), Handbook of sport psychology (3rd ed., pp. 352–378). John Wiley & Sons, Inc.

[ref40] Quatromoni P. A. (2017). A tale of two runners: a case report of athletes’ experiences with eating disorders in college. J. Acad. Nutr. Diet. 117, 21–31. doi: 10.1016/j.jand.2016.09.032, PMID: 28010854

[ref41] Rauh M. J. Nichols J. F. Barrack M. T. (2010). Relationships among injury and disordered eating, menstrual dysfunction, and low bone mineral density in high school athletes: a prospective study. J. Athl. Train. 45, 243–252. doi: 10.4085/1062-6050-45.3.243, PMID: 20446837 PMC2865962

[ref42] Recio-Barbero M. Fuertes-Soriano S. Cabezas-Garduño J. López-Atanes M. Peña-Rotella A. Sáenz-Herrero M. (2019). Delayed diagnosis of an eating disorder in a male patient with superior mesenteric artery syndrome: results from a case study. Front. Psych. 10:731. doi: 10.3389/fpsyt.2019.00731 PMC6803474 31681041

[ref43] Sato Y. Fukudo S. (2015). Gastrointestinal symptoms and disorders in patients with eating disorders. Clin. J. Gastroenterol. 8, 255–263. doi: 10.1007/s12328-015-0611-x, PMID: 26499370

[ref44] Singh S. Contrucci A. L. (2023). Superior mesenteric artery syndrome and anorexia nervosa: a case report. J. Med. Case Rep. 17:459. doi: 10.1186/s13256-023-04168-6 37924161 PMC10625252

[ref45] Sundgot-Borgen J. Garthe I. (2011). Elite athletes in aesthetic and Olympic weight-class sports and the challenge of body weight and body compositions. J. Sports Sci. 29, S101–S114. doi: 10.1080/02640414.2011.565783, PMID: 21500080

[ref46] Sundgot-Borgen J. Torstveit M. K. (2004). Prevalence of eating disorders in elite athletes is higher than in the general population. Clin. J. Sport Med. 14, 25–32. doi: 10.1097/00042752-200401000-00005, PMID: 14712163

[ref47] Tan J. O. A. Calitri R. Bloodworth A. McNamee M. J. (2016). Understanding eating disorders in elite gymnastics. Clin. Sports Med. 35, 275–292. doi: 10.1016/j.csm.2015.10.002, PMID: 26832977

[ref9011] Temme K. E. Hoch A. Z. (2013). Recognition and rehabilitation of the female athlete triad/tetrad: A multidisciplinary approach. Curr Sports Med Rep, 12, 190–199. doi: 10.1249/JSR.0b013e318296190b 23669090

[ref48] Wells K. R. Jeacocke N. A. Appaneal R. Smith H. D. Vlahovich N. Burke L. M.. (2020). The Australian Institute of Sport (AIS) and National Eating Disorders Collaboration (NEDC) position statement on disordered eating in high performance sport. Br. J. Sports Med. 54, 1247–1258. doi: 10.1136/bjsports-2019-101813, PMID: 32661127 PMC7588409

[ref9012] West S. Monteyne A. J. van der Heijden I. Stephens F. B. Wall B. T. (2023). Nutritional considerations for the vegan athlete. Adv Nutr (Bethesda, Md.), 14, 774–795. doi: 10.1016/j.advnut.2023.04.012 PMC10334161 37127187

[ref49] Winston A. P. (2012). The clinical biochemistry of anorexia nervosa. Ann. Clin. Biochem. 49, 132–143. doi: 10.1258/acb.2011.011185, PMID: 22349551

[ref9013] Woodruff K. Clark L. Joy E. Summers S. A. Metos J. M. Clark N.. (2020). An interpretive description of women’s experience in coordinated, multidisciplinary treatment for an eating disorder. GQNR, 7:2333393620913271. doi: 10.1177/2333393620913271 32426422 PMC7218325

[ref50] World Health Organization. (2019) International statistical classification of diseases and related health problems 10th revision. Available online at: https://icd.who.int/browse10/2019/en#/F50.0 (Accessed February 5, 2025).

